# Pharmacologic Therapies to Prevent Relapse of Acute Myeloid Leukemia After Allogeneic Hematopoietic Stem Cell Transplantation

**DOI:** 10.3389/fonc.2020.596134

**Published:** 2020-11-02

**Authors:** Ahmad I. Antar, Zaher K. Otrock, Iman Abou Dalle, Jean El-Cheikh, Ali Bazarbachi

**Affiliations:** ^1^ Almoosa Specialist Hospital, Department of Internal Medicine, Division of Hematology-Oncology, Al-Ahsa, Saudi Arabia; ^2^ Department of Hematology and Oncology, Hammoud Hospital University Medical Center, Saida, Lebanon; ^3^ Department of Pathology and Laboratory Medicine, Henry Ford Hospital, Detroit, MI, United States; ^4^ Bone Marrow Transplantation Program, Department of Internal Medicine, American University of Beirut Medical Center, Beirut, Lebanon

**Keywords:** acute myeloid leukemia, stem cell transplantation, allogeneic, relapse, prevention, hypomethylating agents

## Abstract

Relapse is the main cause of mortality in patients with acute myeloid leukemia (AML) after allogeneic hematopoietic stem cell transplantation (allo-HSCT). Adverse cytogenetic or molecular risk factors, as well as refractory disease or persistent measurable residual disease (MRD) at the time of transplantation are associated with an increased risk of recurrence. Salvage therapy for AML relapse after allo-HSCT is often limited to chemotherapy, donor lymphocyte infusions and/or second transplants and is rarely successful. Effective post-transplant preventive intervention in high risk AML may be crucial. The most frequent and promising approach is the use of post-transplant maintenance with hypomethylating agents or with *FLT3* tyrosine kinase inhibitors when the target is present. Moreover, *IDH1/IDH2* inhibitors and *BCL-2* inhibitors in combination with other strategies are promising approaches in the maintenance setting. Here we summarize the current knowledge about the preemptive and prophylactic use of pharmacologic agents after allo-HSCT to prevent relapse of AML.

## Introduction

Allogeneic hematopoietic stem cell transplantation (allo-HSCT) is currently considered the optimal curative treatment option for patients with unfavorable risk acute myeloid leukemia (AML) (1–3). The implementation of non-myeloablative conditioning regimens and the improvement in supportive care has led to decrease in the transplant-related mortality (TRM) and to significant increase in the number of transplant candidates, including older patients and/or those with comorbidities (4, 5). However, reduced-intensity conditioning (RIC) is associated with higher rate of relapse (6). Allo-HSCT is generally recommended when the benefit of relapse reduction outweighs the risk of non-relapse mortality (NRM)/morbidity and this is based on the assessment of cytogenetic and molecular genetic features as well as donor, patient, and transplant-related factors (7–10). This includes intermediate or high-risk cytogenetic/molecular disease groups defined by the 2017 European Leukemia Net (ELN) guidelines, achievement of complete remission (CR) after more than one induction chemotherapy, refractory disease and the presence of pre-transplant measurable residual disease (MRD) positivity (7, 11, 12).

Disease relapse in transplanted patients in first CR (CR1) occurs in 30%–40% of cases and harbors a particular poor prognosis if it occurs in the first 6 months post-transplant (13). Relapse rates are even higher among patients who undergo allo-HSCT beyond CR1 or those with refractory disease (14, 15).

The treatment options for AML patients who relapse after transplant are very limited and highly depend on the patient performance status at the time of relapse (16). Commonly used treatment options for patients who are candidates for intensive therapy are salvage chemotherapy, often associated with donor lymphocyte infusion (DLI), allogeneic stem cell boost, or even second allo-HSCT from the same or different donor (17–24). In contrast, patients who are not eligible for intensive therapy are usually offered low intensity chemotherapy, hypomethylating agents (HMA), targeted therapies, participation in clinical trials, and withdrawal of immunosuppression or supportive care, all aiming at controlling the disease rather than achieving remission (25–27).

Salvage treatments post-allo-HSCT can induce remissions only in a minority of patients (20%) and the 2-year overall survival (OS) rates are usually below 20% (28–30). Alternatively, preventive strategies have been studied to reduce the incidence of relapse including the use of myeloablative conditioning (MAC), prophylactic DLI, graft manipulation, early withdrawal of immunosuppression or intensive surveillance. Intensification of conditioning regimen by using MAC is associated with a lower relapse rate but with higher TRM. Thus, there is no difference in OS when MAC or RIC are used in allo-HSCT for AML (31). Prophylactic DLI is associated with a decrease in the relapse rate at the expense of more graft-versus-host disease (GVHD) and therefore an increased morbidity and mortality (32).

The low efficacy of these strategies to prevent post-transplant relapse led to the introduction of alternative approaches such as prophylactic pharmacological interventions for patients with unfavorable risk, or preemptive strategies for patients with risk of imminent recurrence indicated by MRD positivity by flow cytometry, cytogenetic testing, molecular analysis or loss of donor chimerism. The ideal maintenance agent should target an active driver pathway, such as tyrosine kinase inhibitors (TKIs) targeting *FLT3* (such as sorafenib and midostaurin) or HMA (i.e., azacitidine and decitabine). These agents have an acceptable non-hematologic toxicity with manageable drug–drug interactions. Moreover, they enhance the graft-versus-leukemia effect (GVL) with non-significant effect on GVHD. For instance, *in vitro* and murine studies showed that HMAs has an important immunologic effect after transplantation in expanding circulating T regulatory (Tregs)/natural killer (NK) cells and up-regulating the expression of tumor antigens on leukemic blasts leading to increased GVL effect without increasing the risk of GVHD (33–35). Moreover, the use of *FLT3* inhibitors as maintenance post-transplant is supported by the observation of an anti-leukemic synergism between sorafenib and alloreactive donor cells (36, 37). One recent study demonstrated that sorafenib promotes GVL activity in mice and humans through interleukin-15 production in *FLT3-ITD* leukemia cells (38).

Here, we summarize the clinical data on a number of agents being studied as maintenance/preemptive therapies after allo-HSCT in AML focusing mainly on TKIs (*FLT3* inhibitors) and HMAs (azacitidine and decitabine).

### MRD Assessment

There are two approaches to reduce the risk of frank AML relapse following allo-HSCT, prophylactic and preemptive strategies. Prophylactic strategies are defined as the initiation of treatment in the absence of any measurable disease after transplant. Prophylactic therapy is given to patients with high risk of relapse in the aim to eradicate residual malignant cells which are undetectable by currently available monitoring techniques. In contrast, preemptive strategies are initiated for patients with risk of imminent relapse presenting as any evidence of disease activity at MRD level to prevent frank hematological relapse.

MRD persistence at transplant has been identified as an independent and strong risk factor for post-transplant relapse that can be at least partially overcome by additional intervention such as augmented conditioning (7, 39, 40). Similarly, growing evidence strongly suggests that MRD detection by multi-parametric flow cytometry (MFC), molecular techniques, or chimerism analyses after allo-HSCT may be used as a predictor of imminent relapse (41). These should be part of routine post-transplant follow-up since MRD detection can improve outcomes by guiding subsequent therapy aiming to unleash or enhance the GVL effect (39).

Dynamic MRD monitoring after allo-HSCT may improve outcomes; however, there is a relative paucity of data and lack of clear recommendation on how we should test MRD (frequency, qualitative and/or quantitative, on peripheral blood or bone marrow), when we should react and what could be the best available MRD-directed intervention post-allo HSCT (42).

The main methods for detection of MRD in patients with AML after allo-HSCT are MFC, molecular genetics and chimerism analyses (43). MFC is the standard and most commonly used MRD method to identify residual leukemic cells reaching a sensitivity of 10^−3^ to 10^−5^ (39–44). Several studies have demonstrated a higher risk of relapse in AML patients with positive MRD detected by MFC after transplant compared to those without evidence of MRD (≤ 0.1% leukemia cells) by the same detection method (45, 46). MRD by flow cytometry has many drawbacks including the lack of standardization, its lower sensitivity, the need for high technical expertise to differentiate between leukemic from regenerating bone marrow cells, biological heterogeneity of the leukemic population and the possibility of false negative results related to sample processing, hemodilution, number of events analyzed and immunophenotypic switch (45–47).

Another method of MRD assessment is donor/recipient chimerism analysis that can detect host-derived hematopoiesis based on genomic differences between the recipient and the donor. Decrease in donor chimerism in AML is often associated with disease relapse (48). Sensitivity of chimerism is dependent on the method applied, ranging from only 10^−2^–10^−3^ in the conventional method using fragment analysis of short tandem repeats (STR) or in XY-FISH analysis method in sex-mismatched donor/recipient, to a high sensitivity of 10^−4^–10^−5^ if variant-allele-specific quantitative PCR that can detect small DNA insertions or deletion or evaluation of CD34+ cell subset in AML were used (48–50). In consequence, chimerism analysis should be routinely performed after allo-HSCT on days +30, +100, +270, and +365 in conjunction with other MRD markers and clinical parameters to wisely decide on preemptive intervention (51).

The last method of MRD assessment is molecular analysis. Currently, the most widely applied strategy for molecular MRD monitoring is real-time quantitative PCR (RQ-PCR) which can detect mutated genes, fusion gene transcripts or overexpressed genes and can detect leukemic cells at 10^−6^ sensitivity (42, 43). PCR based methods are characterized by high specificity and sensitivity for leukemic cells detection and low risk of contamination; however, their use depends on identifying pretreatment AML-associated mutation at diagnosis and these molecular targets must be stable while on therapy (52, 53). For instance, some mutations like *NPM1* mutation, *RUNX1-RUNX1T1* and *CBF-MYH11* in core binding factor (CBF) AML are relatively stable during disease course hereby are suitable for PCR MRD monitoring (12). It was recently shown that *NPM1* MRD-positivity at levels >0.1% to >10% beyond Day +60 post allo-HSCT are associated with increased relapse rates and reduced survival. Hence, preemptive interventions are considered for patients with persistent *NPM1* MRD levels at >0.1%–1% and more intervention should be considered if MRD is >10% (54, 55). Persistent CBF-fusion transcripts after allo-HSCT are translated into higher cumulative relapse incidence (RI) and shorter leukemia-free survival (LFS). Thus, preemptive interventions should be considered in case of persistent MRD positivity (>1%) of *RUNX1–RUNX1T1* or *CBFB-MYH11* in two consecutive measurements or if there is >0.5 log increase in the transcripts in repeated analysis (56, 57).

Other mutations such as *FLT3* (ITD and TKD), *RAS*, *IDH1*, *IDH2*, and *MLL-PTD* may theoretically be measurable by MRD detection but are poor MRD markers and have not been integrated into routine care yet, since these mutations are relatively unstable throughout treatment. Moreover, some of these mutations are lost during disease course and treatment due to leukemia clonal evolution (58). As a result, ELN guidelines recommend against using them as single markers (39).

In contrast to the limited frequency (50%) of mutations mentioned above, over-expression of Wilms Tumor 1 (*WT1*) gene is present in almost 90% of patients with AML and can be measured in peripheral blood with better sensitivity and specificity than in bone marrow. *WT1* expression analysis in MRD assessment is recommended by ELN using a standardized and certified ELN assay (59). Several reports showed that persistent high bone marrow or continuous increase in peripheral blood *WT1* transcripts at 3 months post-transplant are associated with higher risk of relapse (60, 61). Conversely, patients with sustained low *WT1* levels after transplant have excellent outcomes (62).

Other emerging technologies like digital-droplet based PCR and next-generation sequencing (NGS) assays are expected to be particularly useful in AML (63–65).

### Hypomethylating Agents as Maintenance Therapy After Allo-HSCT in AML

**Table 1** summaries the studies that use HMA for relapse prevention after allo-HSCT in AML. HMAs are clinically active in AML and myelodysplastic syndromes (MDS) and represent an important new treatment modality, particularly in elderly and/or unfit patients, due to their favorable toxicity profile (77). HMA have significant antitumor activity in relapsed AML patients after allo-HSCT with a 20%–40% CR rate (78, 79). Azacitidine (AZA) which is the first reported DNMT inhibitor, appears to be well tolerated after transplantation. *In vitro* and murine studies showed that AZA has an important immunologic effect after transplantation in expanding circulating Treg cells and up-regulating the expression of tumor antigens on leukemic blasts leading to increased GVL effect without increasing the risk of GVHD (33).

**Table 1:** Studies using HMA for relapse prevention after allo-HSCT in AML.

Reference	HMA	Study design	Number of patients (disease)	Median age, yrs	Schedule	Median starting time	No of cycles median	GVHD incidence	Response
**de Lima et al. (** 66 **)**	AZA	Phase I	45 (AML: 37; MDS: 8)	60 (24–73)	8, 16, 24, 32 and 40 mg/m^2^ d1–5	+40	1–4	Acute GVHD Grades II–III: 27% chronic GVHD: 37%	- 1-yr EFS: 58% - 1-yr OS: 77%
**Platzbecker et al. (** 67 **) RELAZA trial**	AZA	Prospective Preemptive (detection of MRD after transplant)	20 (AML: 17; MDS: 3)	58 (20–74)	75 mg/m^2^ d1–7	+169	4 (1–11)	——	- 16 Patients (80%) responded (increase or stable CD34^+^ with no relapse)
**Oshikawa et al. (** 68 **)**	AZA	Retrospective matched cohort study	10 (AML)	49 (17–65)	30 mg/m^2^ d1–7 + GO 3 mg/m^2^ d 8	+78	1.5 (1–4)	——	- 1-yr OS (70% in AZA-GO group vs. 59.8% in controls) - 1-yr DFS (60% vs. 42.8%)
**Pusic et al. (** 69 **)**	DAC	Prospective dose finding	22 (AML:17; MDS:5)	59 (21–68)	5, 7.5, 10 and 15 mg/m^2^ d1–5	+50 to +100	5 (1–8)	Acute GVHD grades I–II: 27% grades III–IV: 9%	- 2-yr OS: 56% - 2-yr DFS: 48%
**Han et al. (** 70 **)**	DAC	Phase I	16 (AML:5; MDS:11)	49	5 mg/m^2^ d1–5 then individualized	+86	1–4	Chronic GVHD: 12.5%	——–
**Craddock et al. (** 71 **)** **RICAZA trial**	AZA	Prospective	37 (AML)	60 (40–71)	36 mg/m^2^ d1.5	+54	3–10	Acute GVHD grades I–II: 46% grades III–IV: 0%	- 1-yr OS: 81% - 2-yr OS: 49% - 1-yr RFS:57% - 2-yr RFS: 49%
**El Cheikh et al. (** 72 **)**	AZA	Observational	18 (AML:13; MDS:5)	58 (16–65)	32 mg/m^2^ d1–5	+60	16 (1–45)	Acute GVHD ≧̸ grade II: 28%	- 1-yr OS: 70% - 1-yr DFS: 63%
**Platzbecker et al. (** 73 **) RELAZA 2**	AZA	Prospective Phase II Preemptive (detection of MRD after transplant)	53 (AML:48; MDS:5)	59 (52–69)	75 mg/m^2^ d1–7	——	Up to 24	Acute GVHD grade III: 2%	- 1-yr RFS: 46%
**Oran et al. (** 74 **)**	AZA	RCT	187 AML/MDS AZA (93) Ct (94)	57	32 mg/m^2^ d1–5	+42 to +100	4 (1–12)	——	Median RFS: AZA: 2.07 yrs Ct: 1.28 yrs p = 0.43
**de Lima et al. (** 75 **)**	CC-486	Prospective Phase I/II dose finding	30 (AML: 26; MDS:4)	64 (28–80)	150–300 mg d1–7 or d1–14	+42 to +84	9 (1–12)	Acute GVHD grade III: 10%	- 1-yr OS: 86% (7-day cohort) - 1-year OS: 81% (14-day cohort)
**Marini et al. (** 76 **)**	AZA	Retrospective	32 Pro: 21 Pre: 11	Pro: 58 (15–69) Pre: 52 (30–63)	Pro: 32 mg/m^2^ d1–5 Pre: 75 mg/m^2^ d1–5/7	Pro: +116 Pre: +138	Pro: 6 (1–18) Pre: 4 (4–22)	All GVHD:40%	Pro: - 1-yr OS: 100% - 1-yr EFS: 95% Pre: - 1-yr OS: 82% - 1-yr EFS: 54%

HMA, hypomethylating agent; AZA, 5-azacytidine; EFS, event-free survival; OS, overall survival; GO, gemtuzumab ozogamicin; DFS, disease-free survival; DAC, decitabine; RFS, relapse-free survival; RCT, randomized controlled study; pts, patients; Ct, control arm; yrs, years; pro, prophylaxis; pre, preemptive.

### Prophylactic Therapy With HMA After Allo-HSCT

AZA and decitabine have been tested in several prospective and retrospective studies as maintenance therapy to avoid relapse post-allo-HSCT. These early-phase studies generally demonstrated tolerability, feasibility and established the optimal dosage and schedule for future trials (66, 68–72, 74–76). de Lima et al. (66) reported the results of the first phase 1 dose-finding study of maintenance AZA post-transplant in 45 patients with high-risk AML (n = 37) or MDS (n = 8). The investigators examined subcutaneous AZA at different dosing schedule (8, 16, 24, 32, and 40 mg/m^2^). The optimal dose was 32 mg/m^2^ given for 5 consecutive days every 28 days. After a median follow up of 20.5 months, the NRM was 9%. One-year event-free survival (EFS) and 1-year OS were 58% and 77%, respectively. The rates of grade II-III acute GVHD and chronic GVHD were 27% and 37%, respectively. The authors concluded that low dose azacitidine is safe and may prolong OS and EFS in heavily pretreated AML and MDS patients as post-transplant maintenance (66).

In another report by Oshikawa and colleagues (68), AZA plus gemtuzumab ozogamicin (GO) were used in 10 patients with high-risk AML after allo-HSCT. After a median follow-up of 474 days from allo-HSCT, the NRM rate was 10% and the 1-year disease-free survival (DFS) and OS were 60% and 70%, respectively (68).

Furthermore, in a prospective trial by Craddock et al. (71), 37 AML patients received AZA at a median time of 54 days post-transplant and at a dose of 36 mg/m^2^/day for 5 days every 28 days up to 12 months. AZA was well tolerated in the majority of patients. Only 17 patients had grade I–II acute GVHD. Day 100 and 1-year NRM were 0% and 8%, respectively. The 1-year and 2-year OS were 81% and 49%, respectively (71).

Moreover, El-Cheikh and colleagues (72) reported their results of an observational study on AML (n = 13) and MDS (n = 5) patients who received post-transplant reduced dose AZA of 32 mg/m^2^/day for 5 days monthly, for up to five years. At the time of last follow up, 13 patients were still alive in CR, and had full donor chimerism. The 1-year DFS and OS were 63% and 70%, respectively (72).

More recently, MD Anderson Cancer Center group reported the results of first randomized controlled trial (74). In this study, 187 patients with high-risk AML or MDS who were in CR after allo-HSCT received AZA (n = 93) or placebo (n = 94) at a dose of 32 mg/m^2^/day for 5 days for 12 months. However, most of the patients in the AZA arm (74.6%) did not receive the planned 12 cycles of treatment due to relapse, death, toxicity or upon patient’s request. The investigators closed the study early due to slow accrual. Relapse-free survival (RFS) was comparable between both groups; however, stratification by number of AZA cycles administered showed a trend toward improved RFS in patients receiving more AZA therapy cycles (74).

In addition to injectable AZA, an oral formulation of AZA (CC-486) has been recently tested in a phase 1/2 dose-finding study on 30 patients with AML (n = 26) and MDS (n = 4) in CR as maintenance therapy after allo-HSCT (75). The study included 4 dosing schedules of 150-300 mg per day for 7 or 14 days every 28 days for up to 12 cycles. Oral AZA (CC-486) seemed safe and generally well tolerated with only 3 patients (10%) developing grade III acute GVHD. Median OS was not reached after 19 months follow-up and the 1-year OS were 86% and 81% in the 7-day and 14-day dosing cohorts, respectively (75).

Decitabine is another HMA that has been evaluated in the maintenance setting post allo-HSCT. Pusic et al. (69) tested the safety and efficacy of decitabine maintenance after allo-HSCT in 22 patients with AML (n = 17) and MDS (n = 5). Decitabine was given at a dose of 5, 7.5, 10, and 15 mg/m^2^/day for 5 consecutive days every 6 weeks. The toxicity profile was acceptable. Acute GVHD grade I-II and grade III–IV occurred in 27% and 9%, respectively. The 2-year DFS and OS were 48% and 56%, respectively. The investigators concluded that the dose of 10 mg/m^2^ for 5 days every 6 weeks appeared safe and optimal rather than the 15 mg/m^2^ and could be administered after transplant in high-risk patients (69).

In another study, decitabine was evaluated in a phase 1 dose-finding study as maintenance therapy post allo-HSCT in 16 patients with MDS (n = 11) or secondary AML (n = 5) (70). No aggravation of preexisting acute GVHD was observed and mild/moderate chronic GVHD occurred in only 2 patients (12.5%). In conclusion, the investigators considered 5 mg/m^2^/day to be the most appropriate starting dose for decitabine maintenance (70).

### Preemptive Therapy With HMA After Allo-HSCT

MRD-triggered preemptive therapy with HMA is another strategy to avoid relapse of AML after transplant. The German group has tested this concept in 2 prospective studies (67, 73). The first trial was a single-center phase II study of 20 patients with MDS/AML evaluating the administration of AZA preemptively post allo-HSCT after a decrease of CD34+ donor chimerism to <80%, while still in complete hematologic remission (67). All patients received AZA for 4 cycles at a dose of 75 mg/m^2^/day for 7 days. Sixteen-patients (80%) had response with either increasing CD34+ donor chimerism to >80% (n = 10; 50%) or stabilization (n = 6; 30%) with no evidence of relapse. Furthermore, 11 patients (55%) with stable disease or with subsequent drop in donor chimerism to <80% after initial response received a median of 4 (range: 1–11) additional cycles of AZA. Most patients (65%) ultimately developed hematologic relapse but their relapse was delayed by a median of 231 days after the decrease in donor chimerism.

In the second prospective trial (RELAZA-2) (73), 53 AML/MDS patients who developed MRD positivity after transplant (n = 24) or after conventional chemotherapy (n = 29) received AZA at a dose of 75 mg/m^2^/day for 7 days monthly for up to 24 cycles. MRD positivity were defined by a drop of 80% or less in CD34+ donor chimerism or an increase in *NPM1* mutation, *RUNX1-RUNX1T1* and *CBFb–MYH11* >1% in the bone marrow or peripheral blood without evidence of hematological relapse. One-year RFS was 46%, and 26 (49%) patients eventually relapsed. The authors concluded that AZA could be effectively used to prevent or delay hematologic relapse in MRD-positive patients with AML/MDS (73).

Overall, these data clearly show that AML patients can tolerate maintenance therapy after allo-HSCT with HMA (azacitidine or decitabine) albeit at lower doses, with a favorable safety profile and apparently a reduction in the risk of disease relapse after transplant. Moreover, the results of preemptive studies could serve as the basis to design future studies of MRD-guided therapy using HMAs with other targeted therapies, including immuno-modulating agents.

### FLT3 Inhibitors as Maintenance Therapy After Allo-HSCT in AML

**Table 2** summaries the studies that use *FLT3* inhibitors for relapse prevention after allo-HSCT in AML. *FLT3*-internal tandem duplication (ITD) mutation is found in approximately 30% of patients with AML (91, 92). These patients have a high risk of relapse and low cure rates (93, 94). Patients with *FLT3-ITD* mutation also have a higher risk of early relapse after allo-HSCT compared to patients with wild type *FLT3* (38% vs. 28% in Center for International Blood and Marrow Transplant Research (CIBMTR) analysis) (94, 95). Treatment options for patients with *FLT3*-mutated AML who relapse after transplant are limited to chemotherapy, second allo-HSCT, and *FLT3* inhibitors alone or combined with DLI, all of which are rarely effective in the long term, even though, a small fraction of those patients can achieve long-standing responses with sorafenib (22, 96–99). The use of *FLT3* inhibitors as maintenance treatment after allo-HSCT is supported by the observation of an anti-leukemic synergism between sorafenib and allo-reactive donor cells (36, 37). Moreover, marrow aplasia induced by chemotherapy leads to elevated FLT3*-*ligand levels that may increase on-target activity of *FLT3* inhibitors (100–103).

**Table 2:** Studies using FLT3 inhibitors for relapse prevention after allo-HSCT in AML.

Reference	FLT3 Inh	Study design	Patients number	Median age, yrs	Schedule	Response
**Chen et al. (** 80 **)**	Sorafenib	Phase 1 dose-finding	22	54 (20–67)	200–400 mg BID for 12 months	- 2-yr OS: 78% - 2-yr PFS: 72%
**Antar et al. (** 81 **)**	Sorafenib	Retrospective pilot study	6	50 (32–58)	400 mg BID	100% are alive after median follow-up of 16 months
**Brunner et al. (** 82 **)**	Sorafenib	Retrospective 2 arms	80 (Sorafenib: 26; Control: 54)	- Sorafenib: 54.5 (20–74) - Control: 53 (25–72)	200–400 mg BID for 12–24 months	- 2-yr OS: sorafenib (81%), control (62%) (S) - 2-yr PFS: sorafenib (82%), control (53%) (S)
**Battipaglia et al. (** 83, 84 **),**	Sorafenib	Retrospective Multi-center	28 (maintenance: 25, salvage: 3)	45 (16–57)	200–400 mg BID	- 1-yr OS: 89 ± 7% - 1-yr LFS: 91 ± 6% - 2-yr OS: 80 ± 8% - 2-yr PFS: 73 ± 9%
**Bazarbachi et al. (** 85 **)**	Sorafenib	Retrospective EBMT registry-based analysis	462 (Prophylaxis:19; preemptive:9; Control 434)	50 (19–75)	200–800 mg daily	Matched-pair analysis 26 sorafenib pts and 26 controls: - 2-yr LFS: 79% (sorafenib) and 54% (control) (S) - 2-yr OS: 83% (sorafenib) and 62% (control) (S)
**Burchert et al. (** 86 **) SORMAIN trial**	Sorafenib	Phase II prospective RCT	83 Sorafenib: 43, Placebo: 40	54 (18–75)	200–400 mg BID for up to 24 months	- 2-yr RFS: 85% (sorafenib) - 2-yr RFS: 53% (Placebo) (S)
**Xuan et al. (** 87 **)**	Sorafenib	Phase III randomized	202 Sorafenib: 100, Placebo: 102	18–60	400 mg BID	- 2-yr LFS: 81% (sorafenib) - 2-yr LFS: 54% (Placebo) (S) - 2-yr OS: 83% (sorafenib) - 2-yr OS: 72% (Placebo) (S)
**Maziarz et al. (** 88 **) Radius trial**	Midostaurin	Phase II randomized	60 Midostaurin + SOC: 30 Placebo + SOC: 30	18–70	50 mg BID for up to 12 months	- 1.5-yr RFS: 89% (midostaurin + SOC) - 1.5-yr RFS: 76% (Placebo + SOC)
**Schlenk et al. (** 89 **)**	Midostaurin	Phase II prospective	134 (Midostaurin: 75, Control:59)	18–70	50 mg BID for 12 months	Landmark analysis: Better EFS and OS in midostaurin pts (S)
**Sandmaier et al. (** 90 **)**	Quizartinib	Phase 1 Dose finding	13	43 (23–61)	40 mg daily (n = 7) 60 mg daily (n = 6)	- One relapse among 13 patients

Inh, inhibitor; yrs, years; OS, overall survival; PFS, progression-free survival; DFS, disease-free survival; S, significant; LFS, leukemia-free survival; RFS, relapse-free survival; SOC, standard of care.

Sorafenib was the first TKI studied in the setting of post-transplant maintenance therapy in AML with *FLT3*-*ITD* mutation. It showed benefit in survival and improvement of outcomes in a phase I study, several retrospective studies and two randomized studies (80–86, 104). Chen and colleagues (80) reported the results of the first phase I trial on sorafenib after transplant in 22 patients with *FLT3* mutated AML. They found that sorafenib could be safely used after allo-HSCT with a maximum tolerated dose (MTD) of 400 mg twice daily. The 2-year progression-free survival (PFS) was 72% with a corresponding 2-year OS of 78% after allo-HSCT. Our group has reported the results of a pilot study in 6 patients with *FLT3-ITD* AML who received sorafenib (n = 5 maintenance, n = 1 salvage) after transplant. Grade II skin GVHD was observed in 5 of 6 patients shortly after sorafenib initiation, suggesting a possible immunomodulatory effect. Remarkably, all patients were alive after a median follow-up of 16 months and had sustained molecular remission (81). In a single institution observational study, sorafenib maintenance was evaluated in patients with *FLT3-ITD* AML who underwent allo-HSCT in CR1. Patients on sorafenib maintenance (n = 26) had an improved 2-year OS (81% vs. 62%, p = 0.029) and improved PFS (82% vs. 53%, p = 0.008) compared to historical controls (n = 54) (82).

In a multicenter study, single agent sorafenib was used as post-transplant maintenance in 28 adults with *FLT3* positive AML (83, 84). Twenty-five patients were given sorafenib as primary prophylaxis and three patients received it after relapse post allo-HSCT in combination with salvage chemotherapy and were then continued as maintenance after achievement of CR. At a median follow-up of 18 months, 25 patients were in CR with full donor chimerism with 1-year DFS and OS of 91% and 89%, respectively. A recent update of this study after a median follow-up of 40 months further demonstrated promising long-term outcomes with sorafenib maintenance with 2-year PFS and OS of 73% and 80%, respectively.

Recently Bazarbachi and colleagues (85) reported the results of European Society for Blood and Marrow Transplantation (EBMT) registry-based study on 462 allo-grafted *FLT3*-mutated AML patients (*FLT3*-*ITD*-95%) over a median follow-up of 39 months for surviving patients. Among these patients, 28 received post-transplant sorafenib maintenance as prophylactic (n = 19) or preemptive therapy (n = 9), started at a median of 55 days post-transplant (range 1–173 days) and a median dose of 800 mg/day (range 200–800 mg/day). Multivariate analysis showed that maintenance sorafenib significantly decreased RI [hazard ratio (HR) = 0.39; p = 0.05] with improvement in LFS (HR = 0.35; p = 0.01) and OS (HR = 0.36; p = 0.03). A matched-pair analysis was then performed on 52 patients (26 patients in the sorafenib group and 26 in the control group). The 2-year LFS and OS were 79% and 83%, respectively, in the sorafenib group (p = 0.02) vs. 54% and 62%, respectively, in the control group (p = 0.007).

In a recent double-blind prospective trial (SORMAIN) (86), 83 transplanted *FLT3-ITD* adult AML patients were randomized to receive either maintenance sorafenib (n = 43, up to 400 mg twice daily) or placebo (n = 40) started between days 60 and 100 after transplant for up to 24 months. The 2-year RFS was significantly improved in the sorafenib group (85%) compared to the placebo group (53%) (HR = 0.39, 95% CI, 0.18 to 0.85 p = 0.01). Sorafenib was generally well tolerated and the most common grade III–IV adverse events was acute GVHD (20%) in sorafenib group compared to (17%) in the placebo group.

More recently the Chinese group reported the results of a phase III randomized open-label multi-centers trial on 202 *FLT3-ITD* AML adult patients who underwent allo-HSCT (87). The patients received either sorafenib maintenance (n = 100; 400 mg BID) or placebo (n = 102) within 30–60 days post-transplant and for 6 months. After median follow up of 22 months, eleven and 30 patients relapsed in the sorafenib and control groups. The 2-year OS were 83% and 71%, (P = 0.025) and LFS were 81% and 54% (P < 0.001) in the sorafenib and control groups, respectively.

Acute Leukemia Working Party of the EBMT published a very recent clinical practice recommendation on allo-HSCT in AML patients with *FLT3-ITD* (105). The group recommends post-transplant maintenance with sorafenib in all cases except in patients with active acute GVHD. Sorafenib should be started as soon as possible after disease evaluation and MRD assessment at a dose of 400 mg daily in two divided doses and the dose may be increased to 800 mg daily in case of positive MRD and for a minimum of 2 years, depending on tolerance.

Midostaurin is another *FLT3* inhibitor that has activity as single agent in AML harboring *FLT3-ITD* or *FLT3* tyrosine kinase domain (TKD) mutation. It was also evaluated in the maintenance setting. Based on the RATIFY trial (106), midostaurin received FDA approval in combination with 3 + 7 induction chemotherapy for newly diagnosed *FLT3*-mutated AML. However, in this trial midostaurin maintenance was not offered for patients who underwent allo-HSCT.

The RADIUS phase II prospective trial randomized 60 patients with *FLT3*-*ITD* AML to standard of care (n = 30) or midostaurin (n = 30) starting 28–60 days post-transplant (88). The estimated RFS at 18-month was 76% in the standard of care arm compared to 89% in the midostaurin arm (HR = 0.46; 95% CI 0.12–1.86, P = 0.26), corresponding to relapse rates of 24% and 11%, respectively (P = 0.27).

In another phase II prospective study by Schlenk et al. (89) on 284 newly diagnosed *FLT3*-*ITD* AML patients, midostaurin maintenance treatment was also offered for patients receiving allo-HSCT in CR1 (56%). In a landmark analysis in patients who were event-free at day +100 after transplant (n = 116), those who received maintenance therapy within 100 days post-transplant (n = 72) had better EFS and OS (p = 0.004 and p = 0.01, respectively) than patients who did not.

Gilteritinib is another potent inhibitor of FLT3 with activity against *FLT3*-*ITD* and *FLT3*-*TKD*. In the phase 3 ADMIRAL trial, 371 adult patients with relapsed or refractory *FLT3*-mutated AML were randomly assigned in a 2:1 ratio to receive either gilteritinib or salvage chemotherapy. Patients who had a response and proceeded to allo-HSCT continued in the trial and could resume gilteritinib as maintenance therapy. Median OS in gilteritinib arm was 9.3 months compared to 5.6 months in the chemotherapy arm (107). A follow up on long-term survivors was recently presented in ASCO meeting 2020 (108). After 18 months of follow-up, gilteritinib continued to show better OS rates compared to salvage chemotherapy (27% vs. 15%). A total of 63 gilteritinib-treated patients had OS more than 18 months. A higher proportion of patients on gilteritinib achieved remission and underwent allo-HSCT. After a median of 3.5 months, 35 of 63 (56%) patients underwent allo-HSCT; 25 of these 35 patients (71%) received post-transplant gilteritinib maintenance. The authors concluded that the long-term survival in patients receiving gilteritinib is related to ongoing remission, subsequent allo-HSCT, or post-transplant gilteritinib maintenance therapy. Gilteritinib is currently being prospectively tested as maintenance therapy after allo-HSCT in *FLT3-ITD* AML patients in an ongoing randomized, double-blind, placebo-controlled phase III trial (NCT02997202) (109). This study aims to enroll and randomize 346 adult patients with AML in CR1 to receive maintenance therapy with either 120 mg gilteritinib per day or placebo for 24 months.

Quizartinib, another selective and highly potent *FLT3* inhibitor, was also evaluated in a phase I dose-finding and safety study (90). Thirteen adult patients with *FLT3*-*ITD* mutated AML in morphological remission following allo-HSCT received one of two quizartinib dose levels at 40 mg/day (n = 7) and 60 mg/day (n = 6), administered orally for up to 24 months. Around 77% of patients received quizartinib for at least 1 year and preliminary data indicated an acceptable tolerability and a reduced relapse rate compared with historical cohorts with only one (1/13) relapse.

### Future Perspective

Based on the previously discussed trials, introducing single agent AZA as maintenance therapy can generally delay but mostly not prevent relapse after allo-HSCT. Combining AZA with DLI is a promising concept of MRD-guided post-transplant interventions since it reduces disease burden by cytotoxic therapy and reinforce an allo-immune reaction by cellular approach. This concept was evaluated in a phase II study of 30 patients with high-risk AML (n = 20) and MDS (n = 10) who were treated with prophylactic post-transplant AZA followed by escalated doses of DLI. Two-year OS and DFS were both 65.5%. Acute and chronic GVHD were reported in 31.5% and 53% of patients, respectively (110).

Many targeted agents such as isocitrate dehydrogenase (*IDH*) Inhibitors (*IDH1*, ivosidenib; *IDH2*, enasidenib), hedgehog (Hh) inhibitor (glasdegib), and BCL2 inhibitor (venetoclax) in combination therapy have been evaluated and showed encouraging results in relapsed/refractory (R/R) AML or in AML/MDS patients ineligible for intensive chemotherapy (111–117). Both *IDH* inhibitors were approved by the FDA for the treatment of R/R AML. These drugs induce cellular differentiation and may promote an allo-immunologic reaction by antigen upregulation on leukemic cells. This mode of action implies that these agents may have an interesting activity in *IDH*-mutated AML patients as salvage or even as maintenance therapy after transplant (111, 112). Currently, there are several ongoing prospective trials evaluating the role of *IDH* inhibitors in the maintenance setting after transplant in *IDH*-mutated AML (NCT03515512 and NCT03564821). The safety and efficacy of combination venetoclax plus AZA in R/R AML after allo-HSCT has been proven only in case series (113–116). The same combination is being tested in post-transplant AML patients as maintenance therapy (NCT04128501).

Although combination HMA and *FLT3* inhibitors was not investigated in the setting of maintenance therapy after allo-HSCT in AML, this combination has shown efficacy in AML. DiNardo and colleagues reported the results of the combination of venetoclax with low dose AZA in 81 elderly patients; analysis of primary and adaptive resistance was caused by an enrichment of clones harboring activated signaling pathways such as *FLT3* or *RAS* or biallelically perturbing *TP53* which helped in determining the predictors of outcome using this combination therapy (117). And we know from previous studies that combination of AZA plus sorafenib is effective and well tolerated in relapsed/refractory *FLT3-ITD* AML (118). Thus, the combination of *FLT3* inhibitor and HMA seems to be a potential strategy to prevent relapse post-transplant in high risk AML patients and it is worth being investigated.

Hedgehog inhibitor (glasdegib) has recently shown promising results in a randomized phase II study when combined with low-dose cytarabine (LDAC) as compared to LDAC alone in AML/MDS frail patients (119). A single agent glasdegib is being investigated in a phase II study as maintenance therapy following allo-HSCT for high-risk patients (NCT01841333).

Finally, despite maintenance treatment, most of the patients still relapse. Different mechanisms of resistance may emerge. For example, in patients with *FLT3-ITD* mutation, acquisition of point mutations in the *FLT3* drug binding site, or activation of alternative pathways such as mutations of the *NRAS* gene are the most described mechanism of resistance.^91^ Many combinatorial strategies have evolved and probably overcome this resistance such as combination of *FLT3-TKI*s with epigenetic therapy including histone deacetylase inhibitors and HMA, which revealed promising and synergistic antileukemic *in vitro* efficacy mainly by downregulation of the *JAK/STAT* pathway (120).

**Figure 1:**
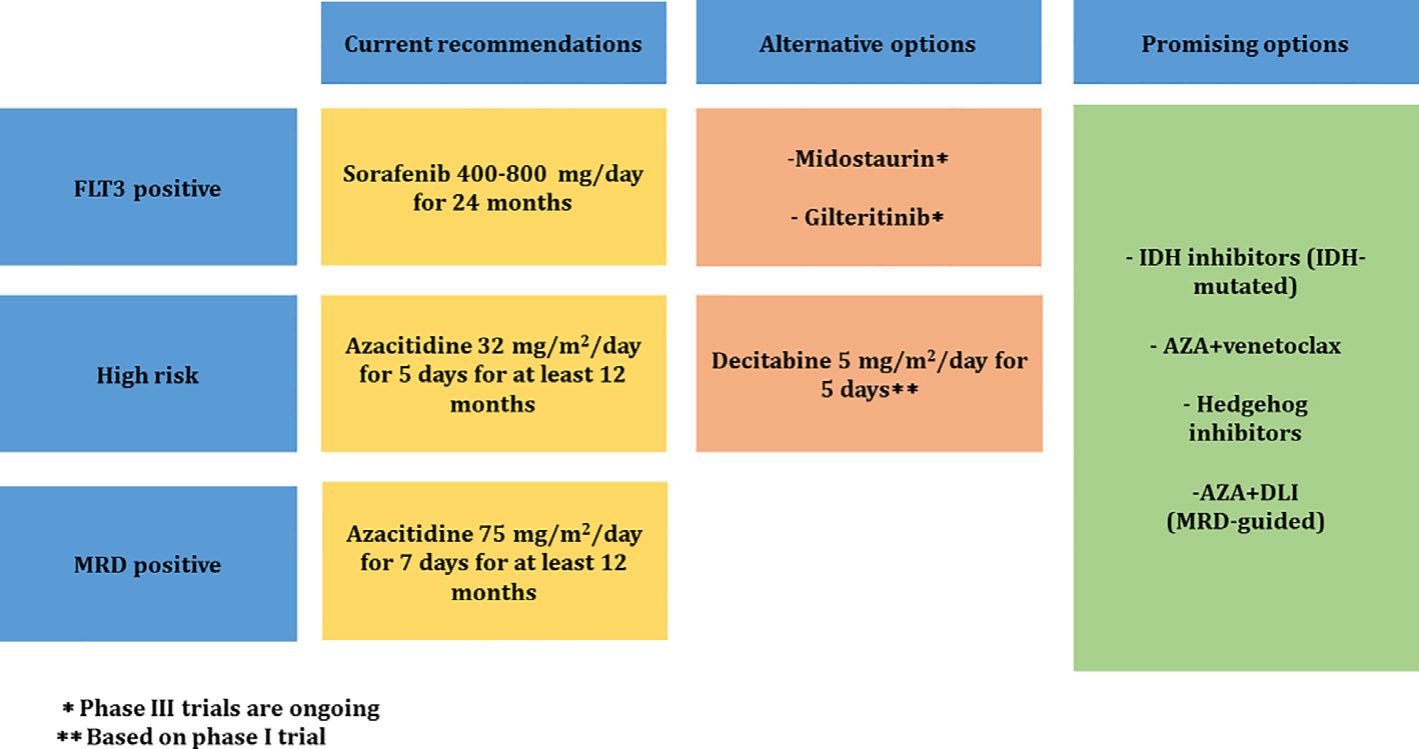
Proposed treatment guidelines to prevent relapse of AML after allo-HSCT. MRD, measurable residual disease; IDH, isocitrate dehydrogenase; AZA, azacitidine; DLI, donor lymphocyte infusion.

**Figure 1** summarizes the treatment guidelines to prevent relapse of AML after allo-HSCT.

### Summary

- MRD measurement using MFC and RQ-PCR methods should be incorporated in the treatment decision process for adult AML patients after transplant.
- MRD will enable to identify high-risk patients to define patients at risk of relapse who would benefit from preemptive approaches with HMA and targeted therapies.
- Azacitidine use as maintenance therapy in high-risk AML and as preemptive MRD-triggered therapy could be considered after transplant for at least 12 months at a dose of 32 mg/m^2^ for 5 days and 75 mg/m^2^ for 7 days, respectively.
- In *FLT3-ITD* AML patients, post-transplant maintenance therapy with sorafenib at a dose 400–800 mg/day in two divided doses should be strongly considered for 24 months.
- Other *FLT3* inhibitors such as midostaurin and gilteritinib are attractive in the maintenance setting and warrant further investigation in larger prospective studies.
- The use of other agents (IDH inhibitors, BCL-2 inhibitors, Hedgehog inhibitors) and combination therapy with DLI are being evaluated and could have a promising result in the post-transplant maintenance setting.

## Author Contributions

AA reviewed the literature and wrote the manuscript. ZO reviewed the literature and wrote the manuscript. IA reviewed the literature and wrote the manuscript. JE-C reviewed the literature and wrote the manuscript. AB reviewed the literature and wrote the manuscript. All authors contributed to the article and approved the submitted version.

## Conflict of Interest

The authors declare that the research was conducted in the absence of any commercial or financial relationships that could be construed as a potential conflict of interest.
